# Incidental Lymphoma Discovered During Surveillance for Low-Grade Upper Tract Urothelial Carcinoma Treated Ureteroscopically: A Case Report Series

**DOI:** 10.1089/cren.2016.0008

**Published:** 2016-02-01

**Authors:** Scott G. Hubosky, Kelly A. Healy, Amar J. Raval, Costas D. Lallas, Joanne Filicko-O'Hara, Demetrius H. Bagley

**Affiliations:** ^1^ Department of Urology, Sidney Kimmel Medical College, Thomas Jefferson University Hospital, Philadelphia, Pennsylvania.; ^2^ Department of Medical Oncology, Sidney Kimmel Medical College, Thomas Jefferson University Hospital, Philadelphia, Pennsylvania.

## Abstract

Two cases of incidentally found follicular lymphoma during surveillance for ureteroscopically treated upper tract urothelial carcinoma with cross-sectional imaging are described. Multiple independent primary malignancies should be considered in this population.

## Introduction

Conservative endoscopic management for upper tract urothelial carcinoma (UTUC) is attractive in carefully selected patients, particularly those with low-grade and relatively low-volume disease. Cross-sectional imaging at regular intervals is recommended because of the potential for local advancement and/or metastatic disease. Given the age demographic for patients with sporadic UTUC, unrelated primary malignancies must be considered especially when imaging shows lymphadenopathy (LAD). We present two cases of primary low-grade UTUC managed endoscopically and both were found to have new onset LAD on CT scan, which ultimately were both diagnosed as unrelated primary follicular lymphomas (FLs). Correct diagnosis is essential to differentiate metastatic disease from multiple independent primary malignancies to allow appropriate treatment.

## Case Report 1

**FIG. 1.**
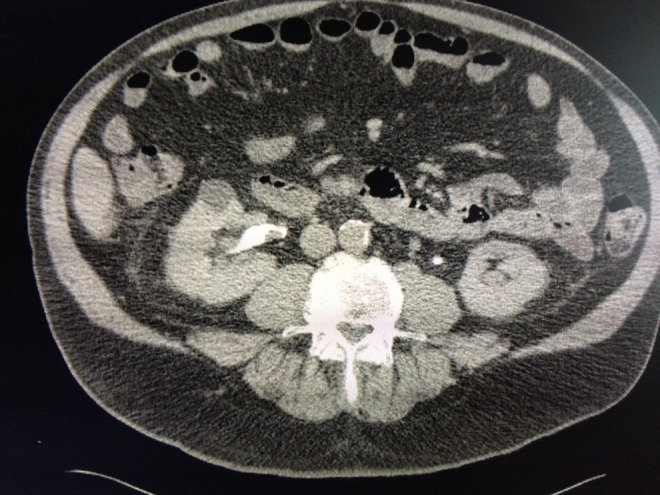
Axial view of CT urogram demonstrates a 1.5 cm filling defect in the right renal pelvis.

A 72-year-old Caucasian woman presented with a 5-month history of intermittent gross hematuria and right flank pain. She had no history of cancer and reported a 29-pack-year smoking history. Physical examination demonstrated no palpable lymph nodes or abdominal masses. Her baseline glomerular filtration rate (GFR) was 54 mL/minute/1.73 m^2^. A CT urogram delineated a 1.5 cm right renal pelvic lesion with no evidence of local advancement or LAD in the abdomen or pelvis (Fig. 1).

Cystoscopy showed no bladder lesions and the left retrograde pyelogram was unremarkable. Right ureteroscopy demonstrated a 1.5 cm papillary lesion, which was biopsied with a 2.4F stainless steel flat wire basket. The lesion was treated with the combination neodymium/holmium YAG laser. A ureteral stent was placed for 1 week.

The ureteroscopic biopsy and site-specific cytologies demonstrated low-grade UTUC. All options were discussed with the patient and ureteroscopic treatment and surveillance were recommended given the patient's age, comorbidities, low-grade pathology analysis, and relatively small tumor size. The patient was followed endoscopically for the next 36 months, occasionally finding low-volume, low-grade recurrences, amenable to endoscopic treatment. No evidence of high-grade UTUC was noted during this time on biopsies or site-specific cytologies. The patient developed small high-grade, noninvasive bladder tumors 12 months after initial presentation, mandating intravesical therapy with BCG, which was well tolerated and effective.

**FIG. 2.**
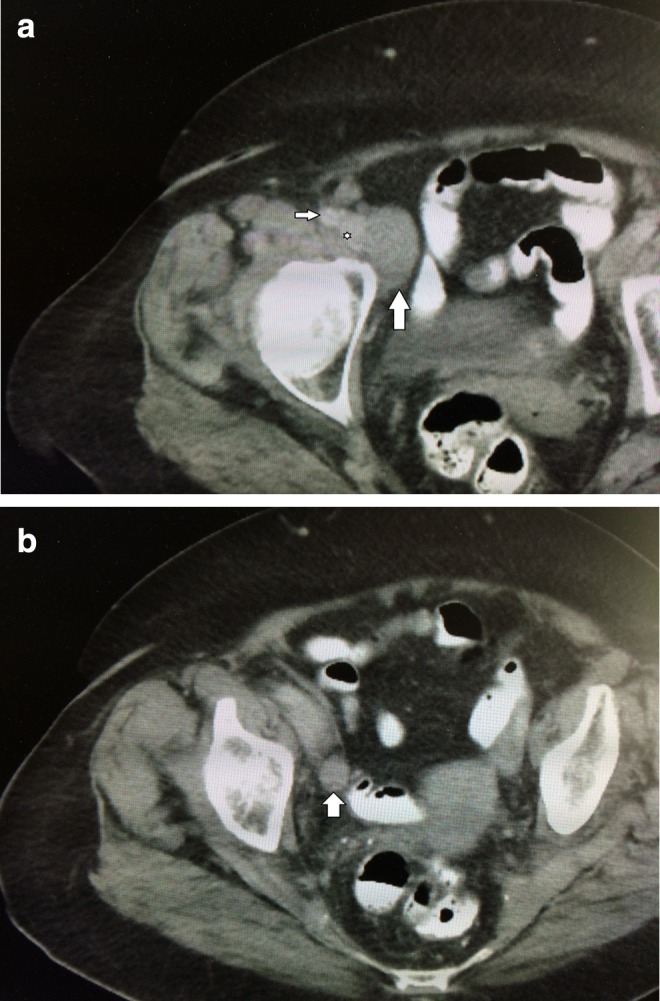
**(a)** CT scan demonstrating 3.3 cm right external iliac node (*vertical arrow*), right external iliac vein (*asterisk*), and right external iliac artery (*horizontal arrow*). **(b)** CT scan showing 3.2 cm right obturator lymph node enlargement (*vertical arrow*).

The patient had cross-sectional imaging yearly with contrast-enhanced CT scans of the abdomen and pelvis without findings of local advancement or metastatic disease. At 42 months from initial presentation, the patient developed significant pain in her right upper leg and complained of a 10-pound weight loss. A CT scan of the chest, abdomen, and pelvis demonstrated new onset of right-sided pelvic LAD with a 3.3 cm right external iliac node (Fig. 2a), 1.6 cm right common iliac node, and 3.2 cm right obturator node (Fig. 2b). The chest, retroperitoneum, and liver were clear. The kidneys demonstrated no evidence of locally advanced disease. A bone scan demonstrated increased uptake in the right femur consistent with malignancy.

Formal lymph node biopsy was deferred by the patient, but a fine needle aspiration of the right external iliac lymph node with flow cytometry demonstrated findings consistent with a B-cell lymphoproliferative neoplasm. The patient's right leg pain spontaneously improved. The presumed diagnosis was an insidious B-cell lymphoma and active surveillance was instituted.

Six months later, a positron emission tomography/computed tomography (PET/CT) scan demonstrated resolution of LAD save for a right axillary node. After formal biopsy, this demonstrated a grade 3A FL. Multiple osseous lesions were now noted but the patient was asymptomatic. Endoscopic surveillance of the right renal unit demonstrated low volumes of recurrent UTUC, which had advanced to high grade. Because of the grade change, a right laparoscopic nephroureterectomy (NU) was performed and pathology analysis demonstrated multifocal high-grade UTUC with pT_a_N_0_ stage with four negative obturator lymph nodes. Within 3 months, PET/CT scan showed progression of osseous lesions but no significant LAD. The patient underwent five cycles of rituximab, cyclophosphamide, doxorubicin, vincristine, and prednisone. Partial remission was noted on follow-up PET/CT scan.

## Case Report 2

**FIG. 3.**
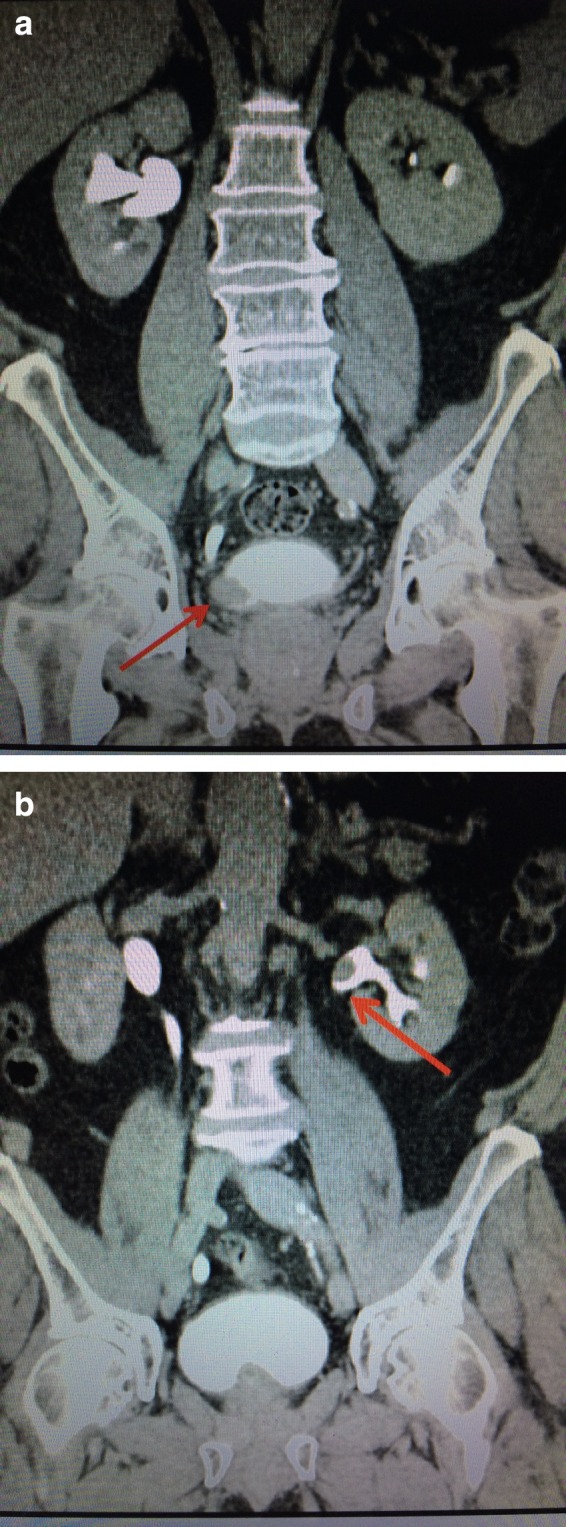
**(a)** Coronal views on CT urogram show 2.6 cm right distal ureteral defect (*red arrow*). **(b)** Simultaneous 1.5 cm left renal pelvic filling defect (*red arrow*).

A 68-year-old Caucasian man presented with a 2-month history of painless gross hematuria. Multiple comorbidities included hypertension, atrial fibrillation on anticoagulation, nephrolithiasis, and a former 30-pack-year smoking history. Personal and family histories were negative for previous cancers. His baseline GFR was 50 mL/minute/1.73 m^2^. A CT urogram demonstrated a 1.5 cm left renal pelvic filling defect and a 2.5 cm right distal ureteral filling defect, but no evidence of locally advanced or metastatic disease was present (Fig. 3a, b). Specifically, no pelvic or retroperitoneal LAD was seen.

Pan-urothelial endoscopic evaluation confirmed a 2.5 cm papillary tumor emanating from the right ureteral orifice and a 1.5 cm left renal pelvic lesion. Owing to its distal location, the right ureteral tumor was treated with transurethral resection. Ureteroscopic biopsies were obtained from the left renal pelvic tumor and the base was then treated with the combination neodymium/holmium YAG laser. Pathology analysis for both lesions showed low-grade UTUC. All options were discussed and the patient chose endoscopic management for his low-grade bilateral UTUC.

Over 18 months, endoscopy showed that the right side remained clear but there were occasional low-volume, low-grade UTUC recurrences on the left, amenable to ureteroscopic treatment. He was found to have a low-risk prostate cancer (clinical T_1a_, Gleason 6, prostate specific antigen 2.1) incidentally after a Holmium laser enucleation of the prostate (HoLEP) for obstructive voiding symptoms. He also developed a high-grade, nonmuscle invasive urothelial carcinoma of the bladder, which responded to a 6-week induction course of BCG.

**FIG. 4.**
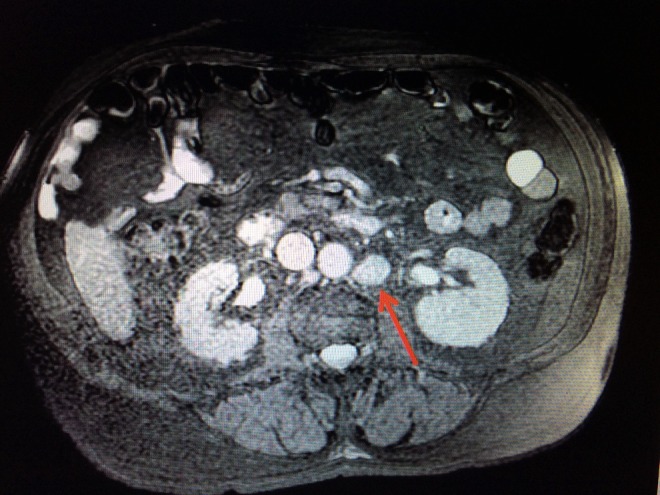
MRI of the abdomen demonstrates an enlarged 2.0 cm left para-aortic lymph node with enhancement after gadolinium (*red arrow*).

Periodic cross-sectional imaging had been performed regularly but a new 2.0 cm left para-aortic lymph node concerning for metastatic disease was discovered on an MRI, 20 months after initial presentation (Fig. 4). CT-guided percutaneous biopsies were nondiagnostic. Subsequently, PET/CT revealed a hypermetabolic 2.6 cm left para-aortic lymph node as well as 3 cm mildly metabolically active para-celiac lymph node. The patient underwent robot-assisted left para-aortic lymph node excision. Pathology analysis showed FL. The patient is on active surveillance and serial imaging demonstrated overall stable disease ∼1 year later.

## Comment

Non-Hodgkin lymphoma (NHL) is the seventh most common cancer diagnosed in the United States with 19.7 new cases per 100,000 persons at risk per year with median age of 66 years at diagnosis.^1^ FL is the second most common B-cell lymphoma behind diffuse large B-cell lymphoma and is the most common indolent form of NHL. In contrast, UTUC is much less common with 2.06 new cases per 100,000 persons at risk per year with median age of 73 years.^2^ Endoscopic treatment of carefully selected patients with low-grade UTUC offers similar survival to extirpative surgery.^3^ Nevertheless, there is a small but persistent risk of progression and current guidelines from both the EUA and NCCN suggest regular follow-up of endoscopically treated UTUC patients with cross-sectional imaging to detect local advancement or metastases.

The challenge with this approach is dealing with the possibility of new onset LAD in an advanced aged population in which multiple primary malignancies may coexist. Nevertheless, after complete endoscopic tumor treatment, we employ yearly cross-sectional imaging. Our practice is to monitor patients with normal creatinine clearance (>60 mL/minute) using CT of the abdomen/pelvis. In patients with mild renal insufficiency (creatinine clearance between 30 and 59 mL/minute), MRI of the abdomen/pelvis is preferred. In patients with chronic creatinine clearance <30 mL/minute, noncontrast CT or MRI can be ordered with the acceptance of lower detection rates for metastatic disease. Alternatively, MRI with gadolinium can be selectively obtained only when the benefit of derived information for the patient is perceived to be greater than the potential risk of nephrogenic systemic fibrosis.

The development of LAD in well-selected low-grade UTUC patients treated endoscopically and sporadic LAD secondary to a new diagnosis of lymphoma are both relatively rare events. The rate of incidental LAD is variable in the radiology literature depending on the definition of an abnormal lymph node radiographically, degree of confirmatory follow-up, and the specific patient population. The prevalence of malignant lymphoma in a healthy Japanese population (59 years median age) undergoing elective cancer surveillance was found to be 0.16% (18/10,659).^4^

Clinically the best way to distinguish LAD as from metastatic UTUC or a new primary lymphoma is to consider the pattern of nodal involvement. In urothelial carcinoma of the bladder or upper tract, metastases typically develop in regional lymph nodes before advancing to the viscera. Matin and colleagues described a mapping study in which patients with UTUC undergoing NU also had wide retroperitoneal lymph node dissections to define tumor landing sites based on laterality and primary tumor location.^5^ Right-sided collecting system and proximal ureteral lesions tended to involve nodes in the paracaval and renal hilar distribution, whereas left-sided collecting system and proximal ureteral lesions demonstrated nodal involvement in the para-aortic and renal hilar areas. Mid and distal ureteral lesions affected retroperitoneal and pelvic lymph node chains almost equally. By contrast, indolent lymphomas, such as FLs, present most often with a palpable or radiographically identified enlarged lymph node in the neck, axilla, abdomen, or groin.

Bone marrow involvement is seen in 40% to 50% of FL cases and demonstrates a patchy pattern of infiltration, leading to frequently false negative bone marrow biopsies. Excisional lymph node biopsy is necessary to diagnose the specific malignant lymphoma because adequate amounts of tissue are required to give histological architecture.

Interestingly, the natural history of FL suggests that as much as 20% to 30% of patients will have transient spontaneous regression of LAD on scans, thus leading to the observation that affected patients might display LAD that “waxes and wanes” with relapses generally within 1 to 2 years.^6,7^ We noted this phenomenon as well as bone marrow involvement in one of our cases. Explanations for spontaneous regression of indolent forms of lymphoma have been proposed based on host immunoregulatory control mechanisms.^8^ Observations of tumor regression following viral or bacterial illness indirectly suggest infection as a stimulus to suppress tumor growth potentially through complement activation. Spontaneous regression of lymphomas in organ transplant patients after discontinuing immunosuppressive therapy suggests that other humoral and/or cellular effector mechanisms may play a role.

These patients demonstrate that a second malignancy can develop during long-term surveillance of low-grade ureteroscopically treated UTUC. Full evaluation including excisional biopsy is necessary to give a diagnosis with new LAD.

## Abbreviations Used

BCG: Bacille Calmette-Guérin
CT: computed tomography
FL: follicular lymphoma
GFR: glomerular filtration rate
LAD: lymphadenopathy
MRI: magnetic resonance imaging
NHL: non-Hodgkin lymphoma
NU: nephroureterectomy
PET/CT: positron emission tomography/computed tomography
UTUC: upper tract urothelial carcinoma
